# Supplementary figures and images for: Towards optimized viral metagenomes for double-stranded and single-stranded DNA viruses from challenging soils
Source: PeerJ. 2019 Jul 4;7:e7265. doi: 10.7717/peerj.7265 (PMC6612421; doi:10.7717/peerj.7265)

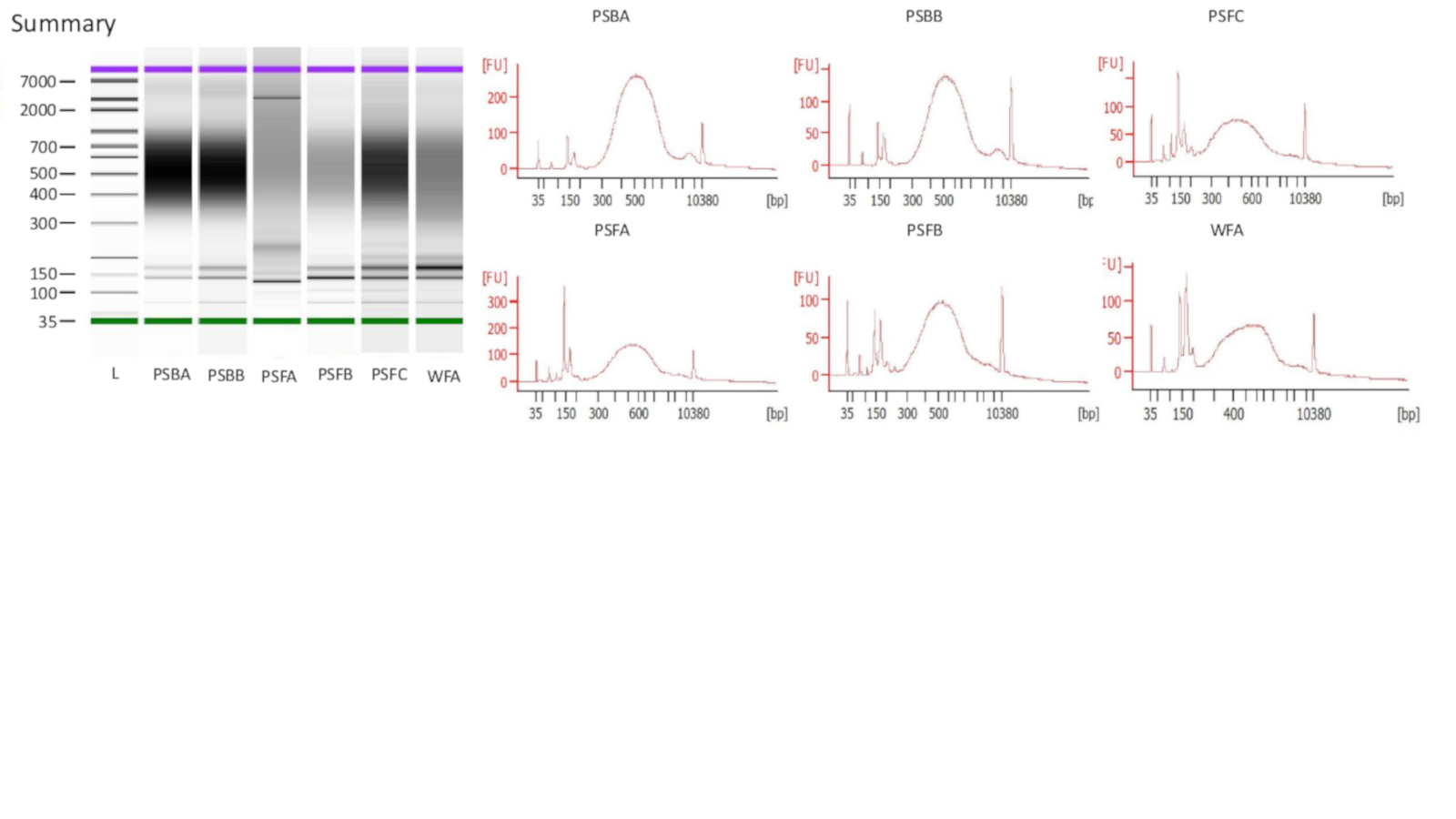

Supplement: Figure S1 — Extracted DNA was run on a Bioanalyzer High Sensitivity DNA Assay for all samples and successful libraries (see methods) are shown. Each sample had 15 PCR cycles. Upper marker designated with purple and lower marker with green. [file peerj-07-7265-s002.png]

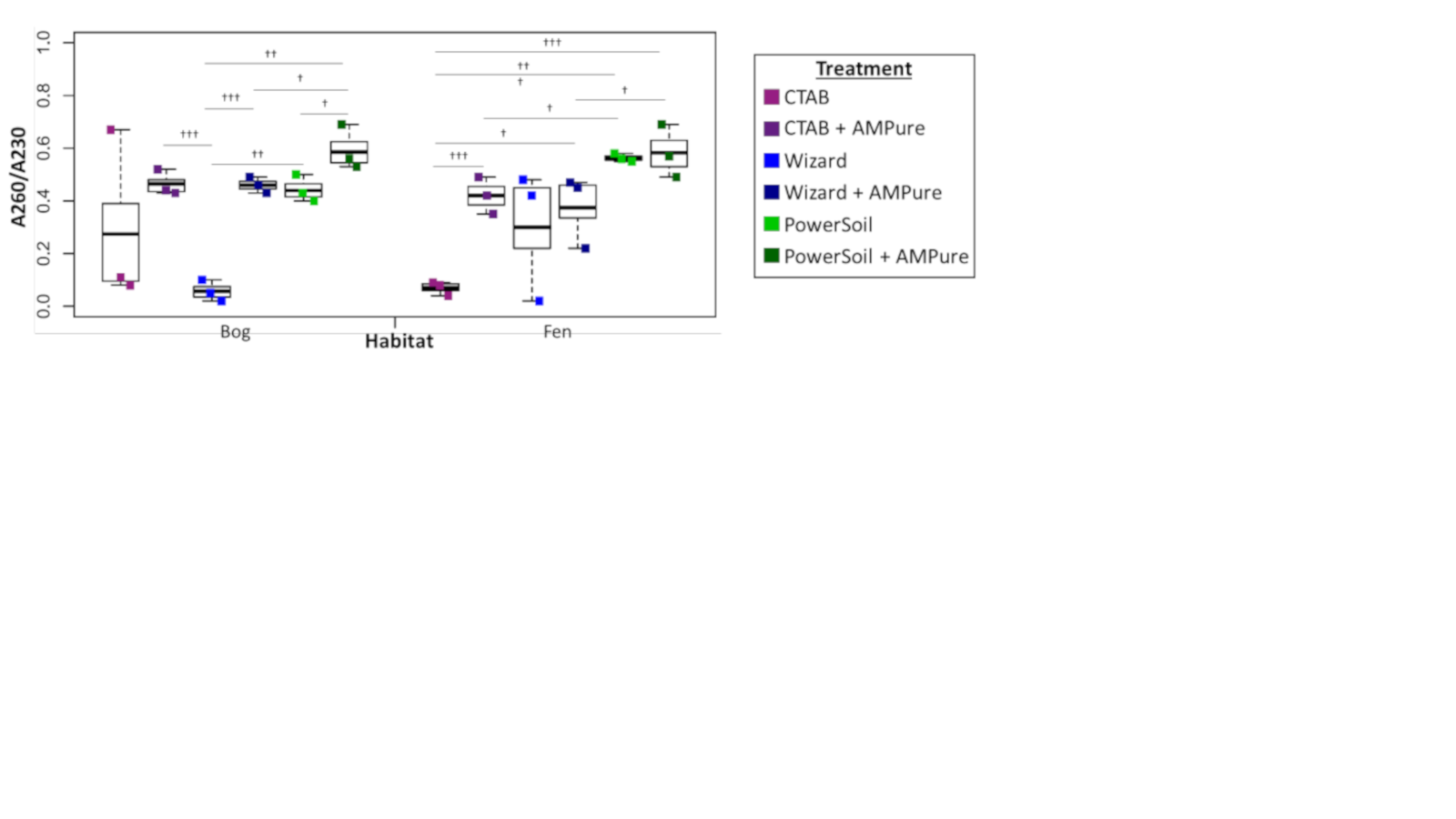

Supplement: Figure S2 — Bog samples are shown on the left of each panel, fen samples on the right. DNA extraction methods are color-coded: purple for CTAB, blue for Wizard, and green for PowerSoil. * denotes significant difference via one-way ANOVA, α 0.05, and Tukey’s test with p-value < 0.05.† denotes significant difference for t test, p-value < 0.05;†† = p-value < 0.01;††† = p-value < 0.001. DNA extract purity via A260/A230 is shown. [file peerj-07-7265-s003.png]

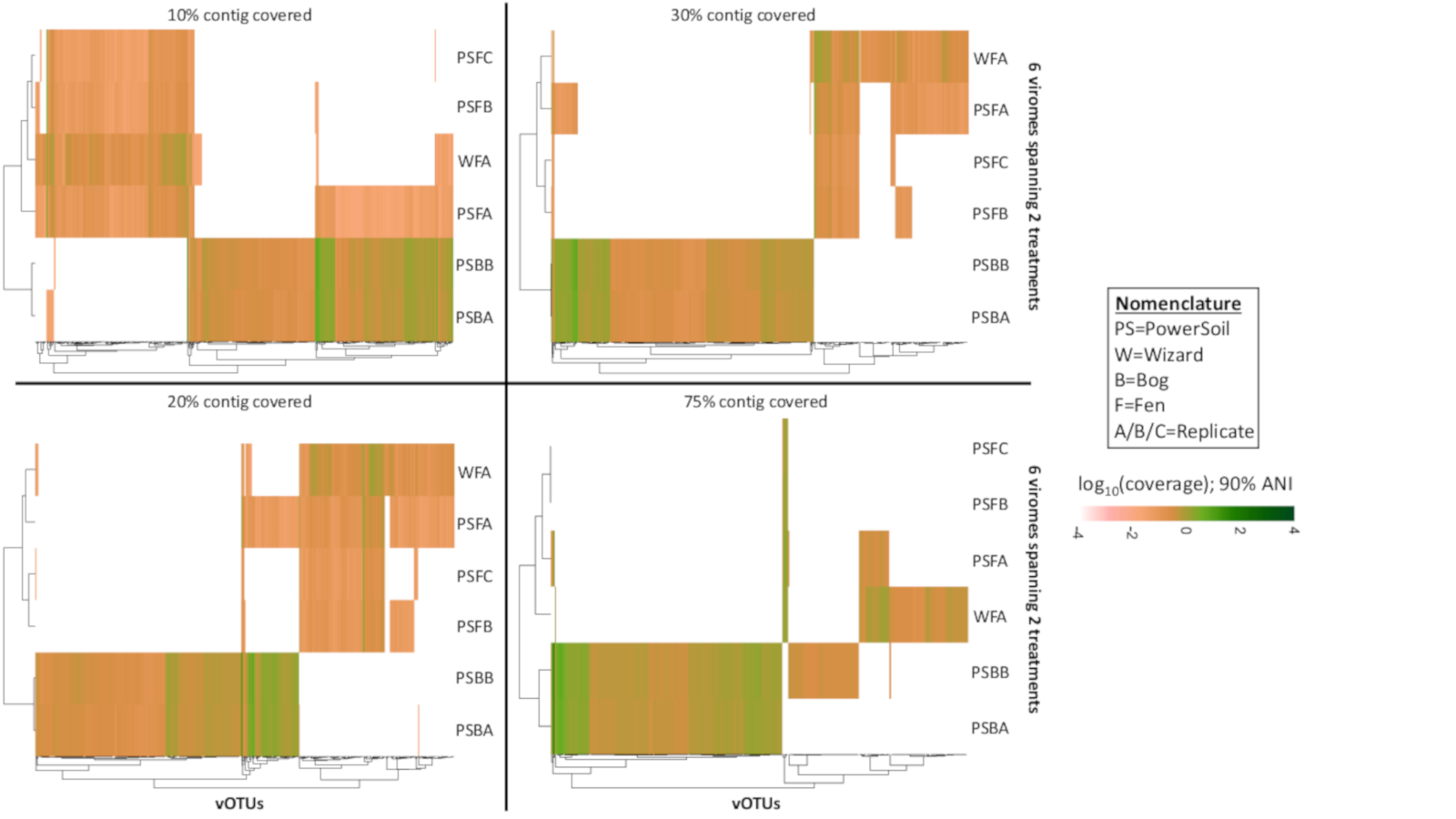

Supplement: Figure S3 — Four heatmaps are shown comparing the relative abundances of the 516 vOTUs with different thresholds on the minimum percentage of genome covered (10%, 20%, 30%, and 75%). The relative abundance was normalized per Gbp of metagenome and log10-transformed. All mapping used a minimum nucleotide identify of 90%. [file peerj-07-7265-s004.png]

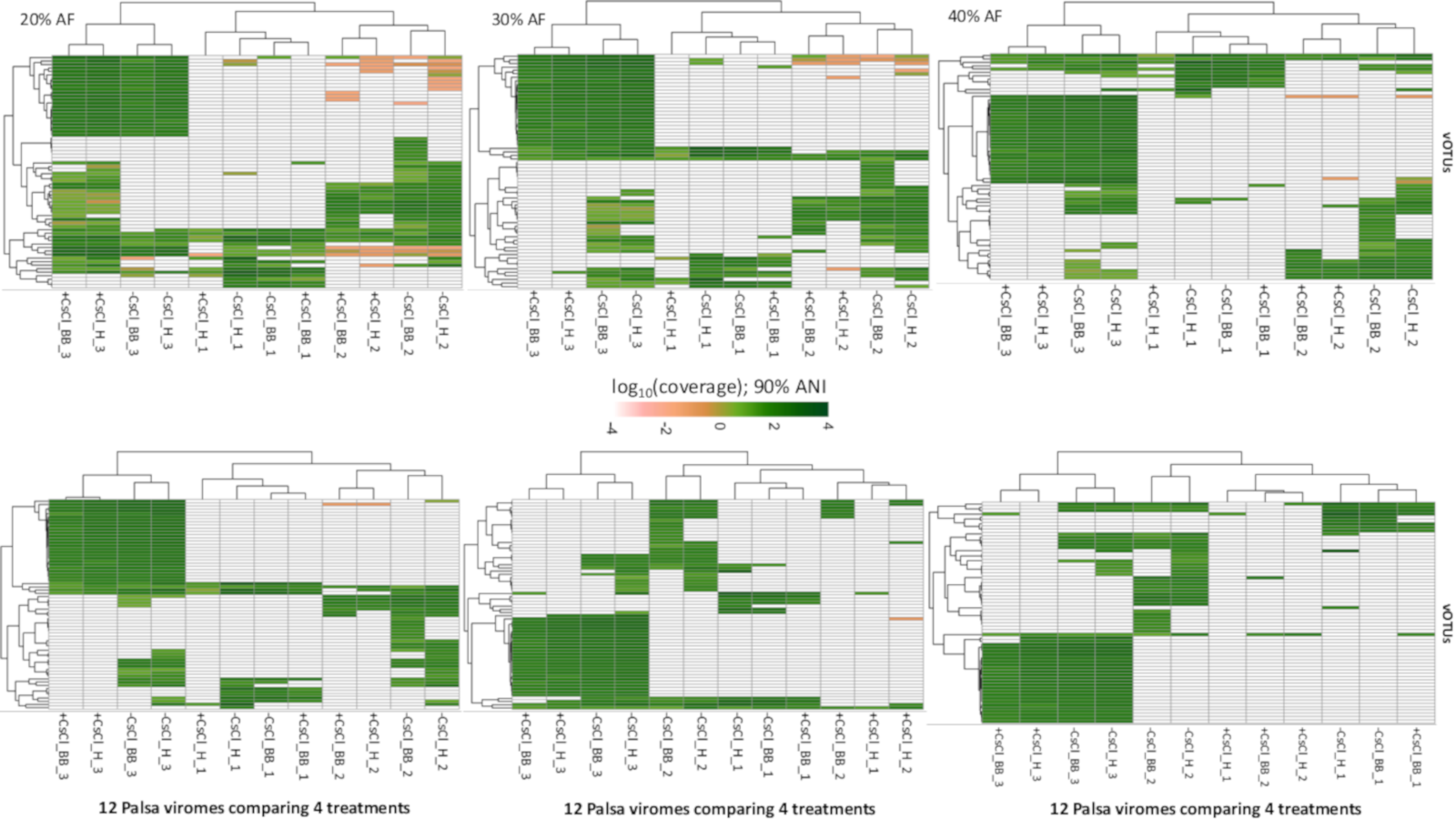

Supplement: Figure S4 — Six heatmaps are shown comparing the relative abundances of the 66 vOTUs with different thresholds on the minimum percentage of genome covered, increasing in increments of 10 (0–60%). The relative abundance was normalized per Gbp of metagenome and log10-transformed. All mapping used a minimum nucleotide identify of 90%. [file peerj-07-7265-s005.png]

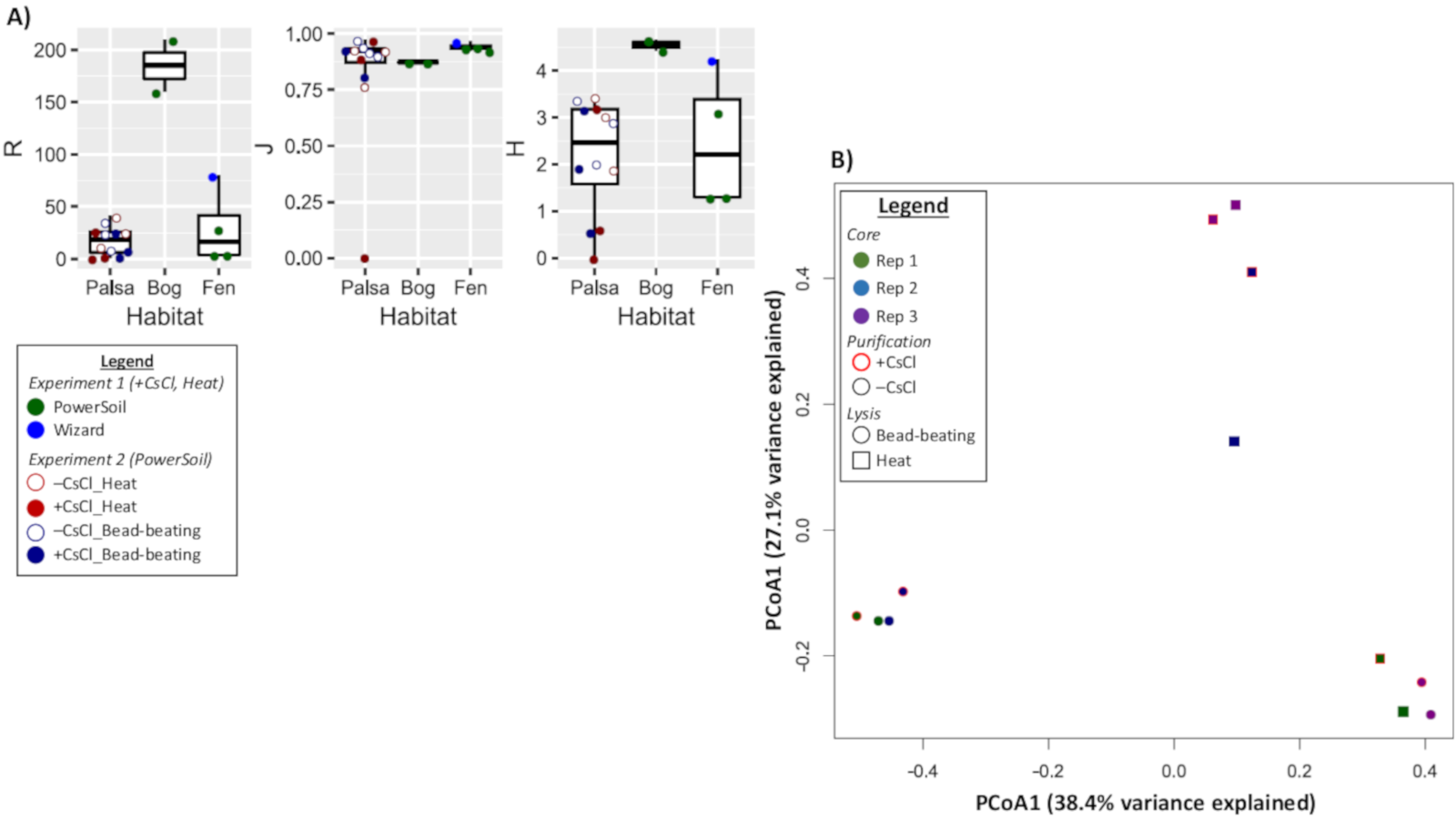

Supplement: Figure S5 — (A)Richness (R),Pielou’s evenness index (J), and Shannon’s Diversity index (H) were calculated for each virome and the viromes are plotted by habitat. Within each habitat the viromes are denoted by a circle, but displayed differently per treatment. For Experiment 1 (bog and fen), viromes are colored green for PowerSoil and blue for Wizard DNA extractions methods. Experiment 2 (palsa) viromes are outlined in red for heat treated samples or blue for bead-beating samples. The marker is filled in for samples that were CsCl purified. (B) A principal coordinate analysis of the viromes by normalized relative abundance of the 66 vOTUs from Experiment 2 based on their Bray-Curtis dissimilarity. Viromes distinguished by soil core, purification (+CsCl outlined in red), and lysis method. [file peerj-07-7265-s006.png]
